# Beyond Huntington’s Disease – Late-Onset Chorea Caused by a Homozygous Variant in ERCC4

**DOI:** 10.1007/s12311-024-01755-1

**Published:** 2024-12-09

**Authors:** Paula C. Barthel, Bertrand Popa, Anne Ebert, Sherif A. Mohamed, Jochen Weishaupt, Julian Conrad

**Affiliations:** 1 Division for Neurodegenerative Diseases, Department of Neurology, Universitaetsmedizin Mannheim, University of Heidelberg, Mannheim, Germany; 2 Department of Neurology, Universitaetsmedizin Mannheim, University of Heidelberg, Mannheim, Germany; 3 Department of Neuroradiology, Universitaetsmedizin Mannheim, University of Heidelberg, Mannheim, Germany; 4 Mannheim Center for Translational Neuroscience (MCTN), Medical Faculty Mannheim, University of Heidelberg, Mannheim, Germany

**Keywords:** Late-onset Chorea, ERCC4, NERD, Genetic, Skin

## Abstract

Genetic alterations in the *ERCC4* gene typically cause Xeroderma pigmentosum and other nucleotide excision repair disorders. Neurologic symptoms are present in some of these patients. In rare cases, *ERCC4*-mutations can manifest with prominent neurologic symptoms. We report a 62-year-old woman who presented with a movement disorder caused by a homozygous pathogenic variant in the *ERCC4* gene. She presented with a hyperkinetic movement disorder (chorea) that affected the distal limbs as well as facial muscles and jaw. There was no ataxia. Extensive clinical evaluation revealed predominantly fronto-parietal and cerebellar atrophy on brain MRI with sparing of the basal ganglia and mesial temporal lobe. Iron and sparse Ca^2+^ deposits were found in the basal ganglia. The detailed neuropsychological evaluation revealed deficits indicating subcortical-prefrontal, subcortical-parietal and frontotemporal dysfunction, without significant impairments in activities of daily living. The audiogram revealed mild age-related hearing impairment, electroneurography was unremarkable without signs of polyneuropathy. The dermatologic examination showed no signs of skin cancer. Knowledge about *ERCC4*-related neurodegeneration is limited and the disease is likely underdiagnosed. Nucleotide Excision Repair Disorder-related neurodegeneration should be considered as a differential diagnosis in patients with adult-onset neurodegenerative disorders, even if dermatologic complications are absent and the family history is negative. The preserved caudate volume in our ERCC4 patient could be a hint towards this rare condition. Treatment is symptomatic. Once the diagnosis is established, patients need to be advised to have regular medical consultations to prevent disease complications such as skin cancer.

## Case report

A 62-year-old woman was referred to the department of neurology for evaluation of a movement disorder of unknown etiology. Huntington’s disease genetic workup had been negative. The patient reported involuntary movements in arms, legs and jaw that started nine years before presentation. She also had trouble finding words for 12 years. Childhood and adolescent development had been unremarkable regarding neurological symptoms. She reported however, to always having been sun-sensitive, resulting in heavy sunburns. There were no further cases of neurologic disorders or history of consanguinity in the family.

Clinical examination showed a hyperkinetic movement disorder that affected the distal limbs as well as head and jaw. Speech was disrupted by sudden short vocalizations and uncoordinated breathing. There was no ataxia.

**Fig. 1:**
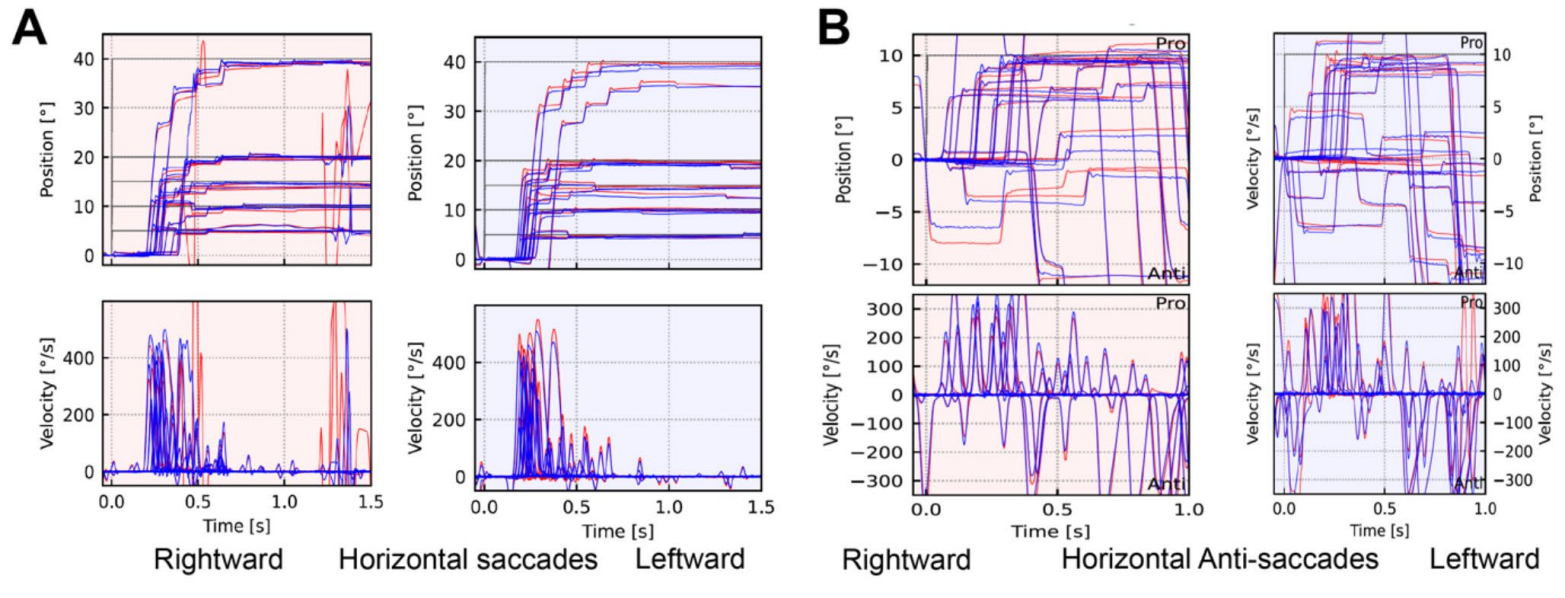
**A** The quantitative ocular motor examination showed saccade hypometria especially at eccentric gaze points (target positions are indicated by black bar). **B** There were frequent anti-saccade errors (pro-saccades) suggesting executive dysfunction. Binocular eye movements recordings were performed using EyeSeeCam Sci2 (EyeSeeTec GmbH, Munich, Germany; https://eyeseetec.de/eyeseecam-sci/). Red right eye, blue left eye

Quantitative ocular motor examination showed saccadic smooth pursuit, hypometric saccades with normal latencies and peak velocities (see Fig. 1A). There were frequent anti-saccade errors (> 50%) suggesting executive dysfunction (Fig. 1B). There was no gaze-evoked nystagmus, vestibulo-ocular reflex and verticality perception were unimpaired.

**Fig. 2:**
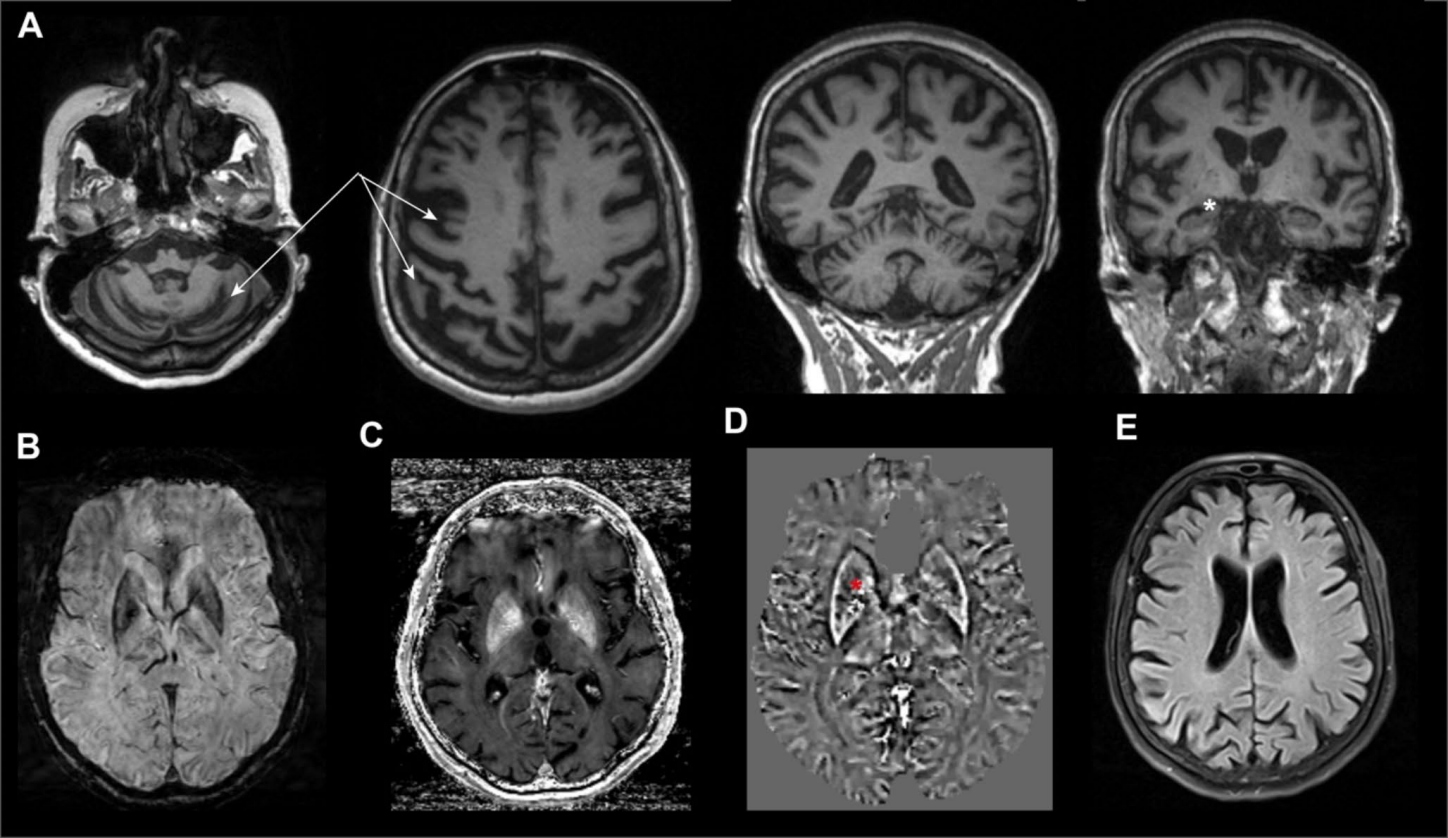
**A** T1w MPRAGE shows pronounced cerebellar and fronto-parietal atrophy (white arrows) with normal hippocampal volume (white asterisk). **B** Susceptibility weighted imaging (SWI) shows regular configuration of the basal ganglia without caudate atrophy. **C** R2* mapping and **D** quantitative susceptibility mapping revealed iron deposits in a rim-like fashion in the putamen and in the internal segment of the globus pallidus with minor diamagnetic signal (i.e., Ca^2+ −^signal; red asterisk). **E** FLAIR imaging shows no evidence of white matter pathology

Brain MRI showed a particularly pronounced fronto-parietal and cerebellar atrophy with relative sparing of the mesial temporal lobe (see Fig. 2A). Additionally, iron deposition was observed in the basal ganglia (Fig. 2B, C). Quantitative susceptibility mapping showed a paramagnetic rim (i.e. iron) in the putamen and mixed signal of paramagnetic and diamagnetic substances (e.g., Ca^2+^) in the internal part of the globus pallidus (2D). The FLAIR sequence showed normal appearing white matter (Fig. 2E).

The serum neurofilament light chain (NfL) levels, as an unspecific marker for neurodegeneration, were borderline normal: NfL 44pg/ml (standard value: < 45 pg/ml). The detailed neuropsychological evaluation revealed deficits in working memory, executive deficits and psychomotor slowing, pointing towards a degeneration of subcortico-fronto-temporo-parietal networks. An extended genetic workup was performed, including a *JPH3* repeat analysis and molecular genetic diagnostic which included *ADCY5*, *ATM*, *CAMK4*, *FRRS1L*, *FTL*, *GM2A*, *GNAO1*, *NKX2-1*, *OPA3*, *PDE2A*, *PDE10A*, *PDHA1*, *PRNP*, *RNF216*, *VAMP2*, *VPS13A*, *XK* and *ERCC4*. This revealed a homozygotic variant in gene *ERCC4* (c.2395 C > T; p.Arg799Trp), previously reported from other Nucleotide Excision Repair Disorder (NERD) patients.

According to the protocol for surveillance in *ERCC4*-associated xeroderma pigmentosum, dermatologic examination and a tone audiogram were performed. The latter showed mildly age-related impaired hearing. There was no evidence of melanoma. Routine dermatologic, neurologic, ophthalmologic and ENT consultations were recommended [1].

The patient reported subjective improvement under treatment with tiapride. She was relieved that a proper diagnosis could be established.

## Discussion

Late-onset chorea has a broad differential diagnosis. The insidious onset and slow progression in this case led to the suspicion of a neurodegenerative disorder. Based on the clinical presentation of our patient, we chose a multigene panel provided by the regional referral center for genetic testing. This included the most common genes associated with choreatic movement disorders, based on genome databases, epidemiological data, the newest scientific evidence and previous described cases. The coding regions as well as the adjacent intron regions and other non-coding, disease-relevant regions were enriched using in-solution hybridization technology and subsequently analyzed by high-throughput sequencing. Using this approach, a homozygotic variant in gene *ERCC4* (c.2395 C > T; p.Arg799Trp) was detected. Based on the current evidence, this variant is likely pathogenic according to the ACMG criteria [2]. This finding was sufficient to explain our patient´s symptoms. Therefore, no further genetic work-up was performed. HD phenocopy cases can be caused by several other mutations such as *TBP*, *C9orf72* or others. In fact, *C9orf72* expansions are among the most frequent causes of HD phenocopy presentations [3]. We cannot ultimately rule out the possibility that additional mutations could also be present in our patient. However, the clinical phenotype with chorea and history of sun sensitivity suggests that the ERCC4 mutation is causally linked to the clinical phenotype.

*ERCC4* mutations are a particularly rare cause of late onset chorea. Usually, *ERCC4* mutations are associated with Xeroderma Pigmentosum group F (XP-F) or other Nucleotide Excision Repair Disorders (NERD), like Cockayne syndrome, Fanconi anemia and XFE progeria [4].

The gene *ERCC4* (ERCC excision repair 4, endonuclease catalytic subunit) at chromosome 16p13.1-p13.2 codes for a protein that forms complex with ERCC1 (ERCC excision repair 1, endonuclease non-catalytic subunit). This protein complex functions as an endonuclease in the nucleotide excision repair (NER) pathway. It is important for preserving DNA integrity and preventing DNA-damage [5, 6]. XP- patients are extremely sensitive to sunlight and prone to skin cancer [5]. Mutations in *ERCC4* are rare, causing approximately 2% of all XP-cases. Generally, neurologic symptoms in XP are common, affecting up to 25% of all XP patients. Possible neurological symptoms include axonal (or mixed) neuropathy with diminished deep tendon stretch reflexes, progressive sensorineural hearing loss, microcephaly, brain atrophy, progressive cognitive decline and ataxia [1]. The occurrence of neurodegeneration is a negative prognostic factor and leads to reduced life expectancy [7]. In XP-F-patients, neurologic impairment is not necessarily present but, if occurring, usually has a late onset in adulthood. This is in contrast to other types of XP which usually start at a younger age [1]. Patients with XP-F tend to have a lower risk for skin cancer compared with other types of XP [8]. Taken together, neurologic symptoms in NERD, especially in XP, are common but usually not the most prominent symptom. However, in rare cases, *ERCC4* mutations may manifest with a purely neurologic phenotype. A recent study investigated patients with biallelic variants in NER genes who presented with a neurological symptoms [9]. Screening 3543 neurological cases, they found 13 patients with biallelic variants in either *ERCC4* (*n* = 8), *ERCC2* (*n* = 4) or *XP-A* (*n* = 1). These patients had adult-onset ataxia, dementia, chorea, neuropathy or spasticity. None of the patients had skin cancer. The authors proposed to introduce NERD_ND_ as adult-onset neurodegeneration within the spectrum of NERD. According to their new nomenclature, they refer to *ERCC4*-related neurodegeneration as NERD_ND_ -*ERCC4*.

Due to the rarity of the disease XP, and even more specific, *ERCC4* mutations, data about the disease prevalence is limited. XP in general affects approximately 1 in 1,000,000 people in the United States and Europe. The subtype XP-F, caused by mutations in the *ERCC4* gene, accounts for 2% of all XP cases [1]. A study that used big data to estimate the prevalence of XP in the US population identified 156 different XP associated mutations in four genome databases [10]. The total allele frequency for the 65 mutations where more information was available, was 1.13%. ERCC4 was among the genes with the most frequent mutations at 0.457% allele frequency. The *ERCC4* p.Arg799Trp mutation seen in our patient is among the most common mutations in the caucasian population. The allele frequency is 4.48 × 10^− 4^ [10]. They occur either as compound heterozygous or homozygous pathogenic variants. There are at least three other XP-F patients described in the literature with the same homozygous pathogenic variant [11, 12].

A few case reports on patients with NERD_ND_ -*ERCC4* have been reported in the literature [12–18]. Clinical characteristics are summarized in Table 1. Compared with these other cases, our patient showed some similarities but also differences. Chorea was the dominant symptom in our patient, but there was no ataxia despite profound cerebellar atrophy. Saccade hypometria was the only purely cerebellar sign detected. Our patient had minor age-related hearing loss. Her neuropsychologic function was affected, but she had no manifest dementia and was able to live independently.

**Table 1:** Clinical and paraclinical characteristics of NERD_ND_ -ERCC4 patients

#Patient, sex, origin	Age at onset	ERCC4-Mutations	Family history	Neurologic symptoms	Cognitive/Psychiatric symptoms	Dermatologic symptoms	Tumor	Additional symptoms	Cerebral imaging	Ref.
#1, f, caucasian	50y	p.Arg799Trp/p.Arg799Trp	Neg	Chorea, saccadic smooth pursuit, hypometric saccades	Subcortical-prefrontal, subcortical-parietal/ frontotemporal dysfunction, without significant impairments in activities of daily living	Photosensitivity	-	Minor age-related hearing loss	Pronounced fronto-parietal-/ cerebellar atrophy; iron-/ Ca2 + deposition in basal ganglia	Present case
#2, m, japanese	44y	c.1608_1617del10/p.Arg454Trp	NA	Ataxia, chorea	Mental retardation	Freckles, mild photosensitivity	BDC		Cortical and subcortical atrophy	[11, 17]
#3, m, caucasian	47y	p.Arg799Trp/p.Arg799Trp	Father: sun sensitivity	Ataxia, dysarthria, chorea, saccadic dysmetria, PNP	Deficiencies in attention/concentration/recall, visuospatial/constructional deficits	Photosensitivity, pigmented macules	8BCC, 9SCC, 1KA	Gonadal dysplasia	Cortical and cerebellar atrophy	[11, 13]
#4, f, caucasian	30y	c.580_584 + 1delCCAAGG/p.Arg799Trp	NA	Ataxia, hypometric saccades, chorea	Mild subcortical frontal profile	Freckles, mild photosensitivity	-		Isolated cerebellar atrophy	[11]
#5, m, caucasian	40y	p.Arg799Trp/p.Arg799Trp	NA	Ataxia, dysarthria, saccadic pursuit, bilateral Babinski signs, spasticity, chorea, PNP	Dysexecutive /working memory dysfunction	Freckles, mild photosensitivity, pigmented macules	-		Global cortical, subcortical and cerebellar atrophy	[11]
#6, f, japanese	30y	p.Trp193Glyfs*6/p.Arg799Trp	Pos (sister of pat. #7)	Ataxia, dysarthria, chorea, saccadic eye movements, hyperreflexia,	-	Photosensitivity	-	Secondary amenorrhea	Cerebellar-/cerebral-/brainstem atrophy	[12]
#7, f, japanese	25y	p.Trp193Glyfs*6/p.Arg799Trp	Pos (sister of pat. #6)	Ataxia, dysarthria, chorea, hyperreflexia,	-	Freckles, photosensitivity	-	Secondary amenorrhea	Cerebellar-/cerebral-/brainstem atrophy	[12]
#8, f, japanese	43y	p.Glu239Gln/p.Glu239Gln	NA	Ataxia, dysarthria, chorea, saccadic eye movements, hyperreflexia	-	Photosensitivity	-		Cerebellar-/cerebral-/brainstem atrophy	[12]
#9, m, japanese	28y	p.Arg799Trp/ p.Arg799Trp	NA	Ataxia, dysarthria, chorea, saccadic eye movements, hyperreflexia, Epilepsy	Dementia	Freckles	-	-	Cerebellar-/cerebral-/brainstem atrophy	[12]
#10, f, caucasian	Mid 20s	p.Arg589Trp/p.Arg799Trp	Neg	Ataxia, dysarthria, PNP, chorea	Progressive cognitive impairment, dementia	Freckles, photosensitivity	-		Severe global atrophy	[16]
#11, f, Ashkenazi Jew	Late teens	p.Arg799Trp/ p.Ser459*	Neg	Ataxia, dysarthria, dystonia, spasticity, chorea	Dementia	Freckles, photosensitivity	> 20 BCC	Neurosensory hearing loss	Global cerebral atrophy, inner table hyperostosis	[16]
#12, m, japanese	44y	p.Arg799Trp/p.Ser805Pro/ p.Gly912Arg	Neg	Ataxia, dysarthria, chorea, PNP	MCI	Freckles, photosensitivity,	1 BCC		Prominent cerebellar atrophy, diffuse cerebral-/brainstem atrophy	[14]
#13, f, caucasian	42y	p.Arg799Trp/p.Trp450Ter	Neg	Ataxia, dysarthria, chorea	Cognitive-behavioral disorders with subcortical symptomatology, executive dysfunction	Freckles, photosensitivity, pigmented macules	-	Bilateral sensory hearing impairment, premature ovarian failure	Massive cortico-subcortical atrophy	[15]

NA = not available; Neg = negative; Pos = positive; PNP = polyneuropathy; BDC = bile duct cancer; BCC = basal cell carcinoma; SCC = squamous cell carcinoma; KA = keratoacanthoma; MCI = Mild cognitive impairment

Unlike other patients, our patient had no clinical or electrophysiological signs of polyneuropathy.

Onset in this case was rather late compared to the previously reported patients. Although most cases reported in the literature experienced the first symptoms in adulthood, this usually occurred earlier in their twenties.

Brain MRI revealed pronounced fronto-parietal and cerebellar atrophy. In the ERCC4 cases published, the degree of atrophy does not correlate with the severity of symptoms [16]. We observed iron deposition in the putamen and sparse calcifications in the internal segment of the globus pallidus using quantitative susceptibility mapping (QSM). This finding is typically observed in primary familial brain calcifications (PFBC) or Cockayne syndrome [18, 19]. As a potential mechanism for clinical symptoms, mineralization with either Fe^2+^ or Ca^2+^ in the pallidum and putamen could potentially disrupt the balance between excitatory and inhibitory pathways in the basal ganglia motor circuits. This in turn could lead to impaired movement control, manifesting as a choreatic movement disorder in our patient. However, it has to be noted that similar types of mineralization can also be detected in asymptomatic individuals. Neurodegeneration in the basal ganglia is an important feature in Huntington’s disease and other movement disorders. In our patient, as an important distinction from Huntington’s disease, there was no caudate atrophy. The imaging features (sparing of the caudate nucleus, basal ganglia Fe^2+^/Ca^2+^ deposition) could help raise suspicion of NERD_ND_ in HD phenocopy cases.

The pathological mechanisms behind neurodegeneration in NERD are poorly understood. NERD have a complex genotype-phenotype spectrum, and the affected genes are involved in various pathways, in addition to the global genome nucleotide excision repair pathway. This includes e.g. posttranslational DNA-modifications and antioxidative function [4, 20]. It is proposed that post-mitotic cells, such as neurons, are especially vulnerable for long-term effects of accumulating DNA-damage due to insufficient repair mechanisms as well as for oxidative stress and mitochondrial dysfunction which could be possible pathomechanisms behind NERD_ND_ [9, 4, 20].

Therapeutic options for patients with ERCC4-related neurodegeneration are limited. At present, no causative treatment is available for NERD_ND_ -ERCC4. Symptomatic treatment includes tetrabenazine or tiapride which substantially decreased the intensity of the choreatic movements in our patient. In addition to symptomatic treatment, regular surveillance especially for skin malignancies and hearing loss are key components in the care of patients with *ERCC4* -related neurodegeneration.

Based on the existing data, it is necessary to introduce NERD_ND_ as adult-onset neurodegeneration within the spectrum of NERD [9]. NERD_ND_ is likely underdiagnosed and should be considered in patients with adult-onset neurodegenerative disorders, especially movement disorders, when more common hereditary and non-hereditary diagnoses are ruled out [9]. It is important to appreciate that NERD_ND_ can be present in absence of dermatologic manifestations and a negative family history. Further research and awareness for NERD_ND_ is necessary to unravel the disease mechanisms, to refine diagnostic procedures and develop causative therapeutic strategies.

## Data Availability

No datasets were generated or analysed during the current study.

## Abbreviations

NfL: Neurofilament Light Chain
ERCC4: Excision repair 4, endonuclease catalytic subunit
XP: Xeroderma Pigmentosum
XP-F: Xeroderma Pigmentosum group F
NERD: Nucleotide Excision Repair Disorders
ERCC1: ERCC excision repair 1, endonuclease non-catalytic subunit
NER: Nucleotide excision repair
ERCC2: ERCC excision repair 2, TFIIH core complex helicase subunit
XP-A: Xeroderma Pigmentosum group A
NERD_ND_: Adult-Onset Neurodegeneration in Nucleotide Excision Repair Disorders
NERD_ND_-ERCC4: ERCC4-related neurodegeneration
